# Volume Four

**Published:** 1883-12

**Authors:** 


### VOLUME FOUR.

The tendency of modern journalism is toward condensation of matter. Short, clearly stated articles are the ones best appreciated. It is therefore our desire, in our next year's work, to follow this method in the presentation of our articles. We beg our contributors to bear this in mind in writing for our pages.

This number contains some controversial matter; differences will arise between honest workers. Beside this element, we still give much thoroughly practical matter.

